# Mixed-thiol functionalised gold nanoparticles for trastuzumab delivery: chemical interaction and photothermal therapeutic potential

**DOI:** 10.1039/d6ra07147c

**Published:** 2026-10-14

**Authors:** Tommaso A. Salamone, Rosanna Mosetti, Annalisa D'Arco, Stefano Lupi, Francesca Petronella, Arianna Avitabile, Flaminia Pompeo, Luciano De Sio, Sofia Marotta, Stella Mrmić, Beatrice Pennacchi, Sara Cerra, Souren Grigorian, Michael Paulus, Maurizio Alimandi, Pankaj Trivedi, Eleni Anastasiadou, Ilaria Fratoddi

**Affiliations:** a Department of Clinical and Molecular Medicine, Sapienza University of Rome Rome 00189 Italy; b Department of Chemistry, Sapienza University of Rome Rome 00185 Italy Tommaso.salamone@uniroma1.it ilaria.fratoddi@uniroma1.it; c Department of Basic and Applied Sciences for Engineering, Sapienza University of Rome 00161 Rome Italy; d Department of Physics, Sapienza University of Rome Rome 00185 Italy; e National Research Council, Department of Chemical Sciences and Materials Technologies, Institute of Crystallography Montelibretti 00010 Italy; f Department of Medico-Surgical Sciences and Biotechnologies, Sapienza University of Rome Latina 04100 Italy; g Institute of Physics, University of Siegen Siegen D-57068 Germany; h Fakultät Physik/DELTA, Technische Universität Dortmund 44221 Dortmund Germany; i Department of Experimental Medicine, Sapienza University of Rome Rome 00161 Italy

## Abstract

Small-sized gold nanoparticles (AuNPs) functionalised with hydrophilic thiols were investigated. Sodium 3-mercapto-1-sulfonate and 2-(diethylamino)ethanethiol hydrochloride were used as stabilising agents in a wet synthesis approach to provide a stable colloidal system. AuNPs were loaded with the antibody trastuzumab (TZ) to evaluate the potential use of the system for photothermal-assisted controlled drug release. Furthermore, to gain a deeper understanding of the chemical interactions occurring at the interface between TZ and the surface thiols of the nanoparticles, FT-IR studies were performed both in the solid state and in suspension, suggesting a strong interaction between the surface thiols and the amidic functional groups of TZ. The photothermal (PT) behaviour of the pristine AuNPs was investigated under continuous-wave laser irradiation at 532 nm, matching the plasmon resonance band of the gold nanoparticles, resulting in an up to 20 °C temperature increase. FT-IR measurements were also carried out after laser irradiation to evaluate the potential for a controlled release of TZ from the nanoparticle surface, suggesting that light irradiation can trigger the release of the drug from the AuNPs surface, with no detrimental effect from the temperature increase. Finally, SAXS studies enabled the investigation of TZ loading and release kinetics *via in situ* studies over 3 hours. This work provided a detailed investigation of the interfacial chemical interactions, also giving a dual potential application of the hybrid, evaluating its use for PT therapy and for PT-assisted controlled release of the antibody.

## Abstract 2

Small-sized hydrophilic gold nanoparticles (AuNPs) loaded with trastuzumab: interfacial interactions and photothermal study.

## Graphical abstract

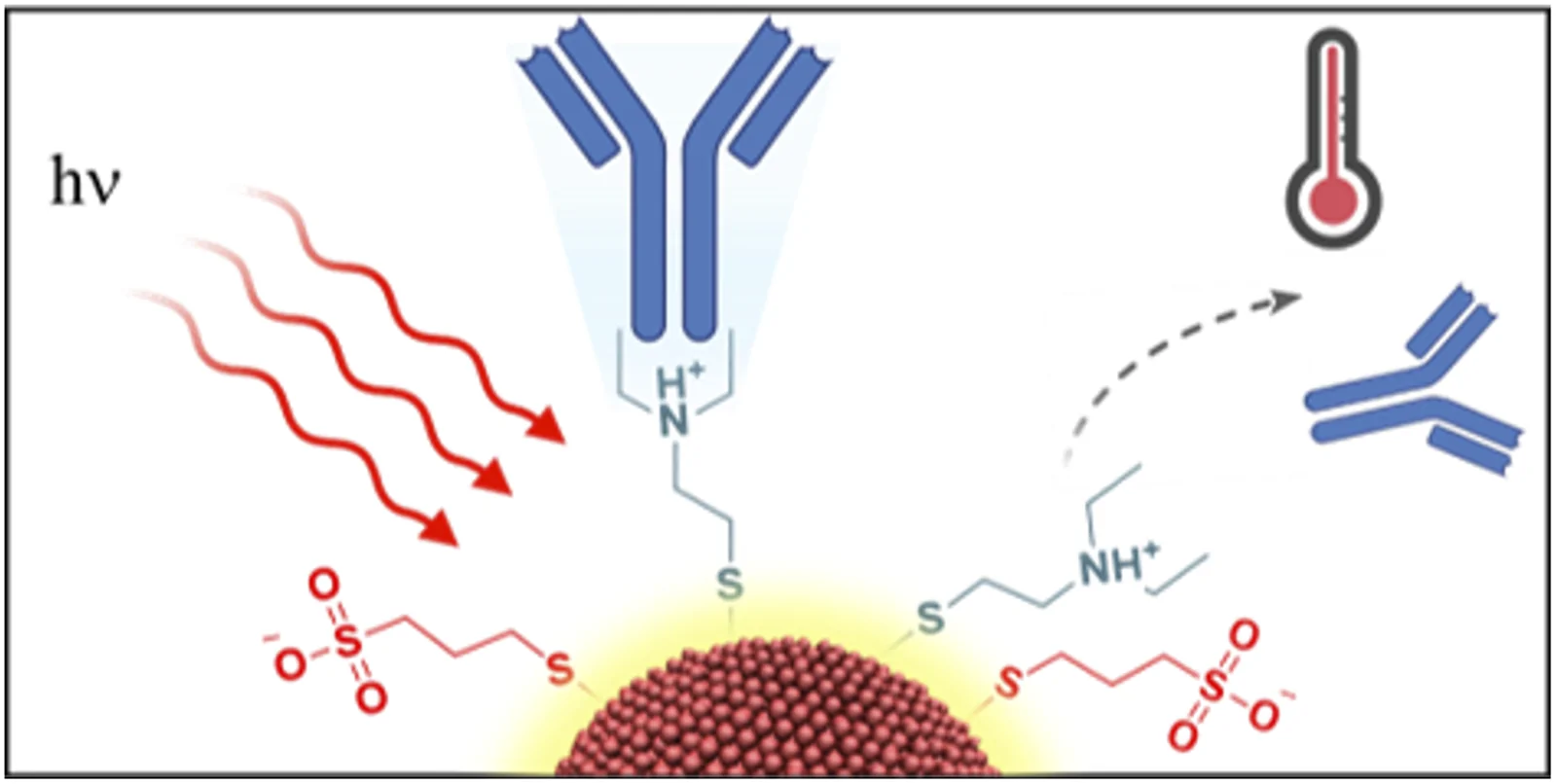

## 1. Introduction

Gold nanoparticles (AuNPs) have been at the forefront of research in different scientific fields, ranging from optoelectronics to medicine.^1^ The peculiar properties derived from their size, shape, and surface chemistry drive the applicative efforts; a crucial aspect of the chemistry of AuNPs is their surface functionalisation.^2^ Over the years, different classes of molecules have been employed as surface functionalising agents for gold nanoparticles, protecting them from aggregation and conferring added functionality to the nanomaterial; including phosphine derivatives, carbenes, surfactants, and thiols.^3–5^ To date, sulphur-based thiols are the most widely used and recognised as “gold standard”, thanks to the stable Au–S covalent bond that ensues during the synthesis, and the wide range of compounds that can be employed as functionalising agents.^6^ The chemistry of the surface ligand influences the overall chemistry of the AuNPs, as mentioned above; in this respect, close attention has been given to the possibility of employing highly hydrophilic gold nanoparticles as carriers for bioactive agents, such as anticancer drugs, antibodies, or oligonucleotides.^7–9^ The advantages of such an approach lie in the enhanced bioavailability of lipophilic drugs when loaded on hydrophilic AuNPs, as well as the exploitation of the Enhanced Permeability and Retention effect, which allows for small (5–100 nm) nanoparticles to accumulate in tumour tissue.^10^

A crucial factor in designing AuNPs for drug delivery and understanding their properties and mechanisms of action is the study of the interaction between the surface functionalising layer and the bioactive agents; non-covalent interactions, exploiting electrostatic forces, van der Waals forces, or other weak interactions, can be exploited to deliver bioactive molecules, also for the controlled release of drugs.^11^

Gold nanoparticles in nanomedicine are also exploited for photothermal therapy (PTT), where irradiation with a laser in the absorption region of the AuNPs causes an increase of the local temperature.^12^ Exploiting this effect has been proven useful to release, in a controlled manner, drugs loaded on the surface of AuNPs.^13^ Further applicative opportunities in the biomedical field include PT cancer ablation,^14,15^ as well as drug and gene delivery.^16–18^

There has been significant interest in nanoparticles and, more broadly, nanomaterials for medical applications, with other nanostructured systems already being approved for clinical use or in clinical trials, such as lipid nanoparticles, liposomes, and polymeric nanoparticles.^19,20^ While these formulations offer advantages in facile synthesis, reproducibility and innate biocompatibility, their surface chemistry cannot be modified, or it can be modified only marginally. In this respect, gold nanoparticles offer a unique advantage in the versatility of surface functionalisation where different strategies can be exploited to improve active targeting, controlled release, and efficient loading of the bioactive payload. Their surface charge, moreover, can be modulated simply by choosing the appropriate functionalising agents.^21,22^

In previous works from our group, a mixed-thiols functionalising layer allowed us to obtain AuNPs exceptionally stable against aggregation. Two thiols were used as ligands, one with a net negative charge, sodium 3-mercaptopropanesulfonate (3MPS), and another one with a net positive charge, 2-(diethylamino)ethane thiol hydrochloride (DEA), yielding AuNPs-3MPS-DEA. This peculiar functionalisation layer allowed us to study this system as a drug delivery platform, both for small anticancer drugs (methotrexate, MTX) and for antibodies (trastuzumab, TZ); in both cases, *in vitro* tests showed the enhanced cytotoxic effect against cancer cells of the loaded nanoplatforms compared to the bioactive agents alone.^23,24^

In this work, we focussed on the structural features at the basis of antibody loading, stabilisation and release from the thiol covered Au surface, a factor which remained insufficiently defined. For potential therapeutic uses, it is of paramount importance to understand the nature of the chemical interactions between functional groups. To do so, we employed spectroscopic techniques such as Fourier Transform Infrared spectroscopy (FT-IR), as well as Small Angle X-ray Scattering (SAXS) to study the kinetics of its loading and release on the surface of the nanoparticles. Lastly, using FT-IR spectroscopy and laser irradiation, we were able to firstly investigate their photothermal behaviour, studying AuNPs and AuNPs-TZ dispersions after irradiation at 532 nm. These studies led us to understand the vibrational behaviour of the protein and the AuNPs, along with their photothermal behaviour, resulting in a laser-driven combined effect: the plasmonic induced TZ release, together with a significant temperature increase. These results highlight the possibility of using such systems as nanomedicine probes, for combined PTT and controlled release.

## 2. Materials and methods

### 2.1 Materials and reagents

Tetrachloroauric(III) acid trihydrate (HAuCl_4_^·^3H_2_O, 99.0%, M.W. 393.83 g mol^−1^), sodium borohydride (NaBH_4_, 99.99%, M.W. 37.83 g mol^−1^), sodium 3-mercapto-1-propanesulfonate (HS(CH_2_)_3_SO_3_Na, 3MPS, 90%, M.W. 178.21 g mol^−1^), 2-(diethylamino)ethanethiol hydrochloride ((C_2_H_5_)_2_NCH_2_CH_2_SH·HCl, DEA, 95%, M.W. 169.72 g mol^−1^), 4-(2-hydroxyethyl)-1-piperazineethanesulfonic acid 0.1 M (C_8_H_18_N_2_O_4_S, HEPES), were used as received from Merck and used without purification. Ultra-pure water (H_2_O_up_, 18.3 MΩ cm) was produced with a Zeneer Power I Scholar-UV (Full Tech Instruments) deionisation/purification apparatus. Cellulose acetate dialysis tubing with molecular weight cut-offs (MWCO) = 12 000 Da (Merck) were used upon pre-conditioning with distilled water for 12 h. Trastuzumab (TZ) in an aqueous solution (21 mg mL^−1^) was provided by San Camillo Hospital in Rome.

### 2.2 Methods

#### 2.2.1 Synthesis of AuNPs-3MPS-DEA

AuNPs-3MPS-DEA were synthesised following a previous report by our group.^23^

#### 2.2.2 Characterisations^23^

UV-Vis (H_2_O): *λ*_max_ = 520 nm; Q2.4 hydrodynamic diameter <2*R*_H_> (DLS, H_2_O): (9 ± 2) nm; yield: 29 ± 8% wt; FTIR (ATR, solid): *ν* = 3060 (w), 3020 (w), 1584 (m), 1472 (s), 807 cm^−1^ (s).

#### 2.2.3 Loading and release of TZ on AuNPs-3MPS-DEA

The loading and release processes were carried out as previously published,^24^ with appropriate modifications necessary for the kinetics studies with SAXS. Briefly, TZ was added to a dispersion of AuNPs in a 1 : 1 weight ratio and gently stirred at +4 °C for 3 hours, subsequently the mixture was centrifuged, and the precipitate resuspended in H_2_O_up_ for further spectroscopic studies.

For the study of the loading kinetics, the same protocol was followed, and the mixture was charged in a glass capillary suitable for SAXS studies and the loading was studied at +4 °C overnight with spectra being recorded every 10 minutes. For the release study, following what was described above, after centrifuging to remove unbound antibody and resuspension, the dispersion in a SAXS glass capillary was measured every 5 minutes overnight at +37 °C.

Loading and release percentages were calculated following our reported methodology, *i.e.*, a Bradford assay was conducted on the supernatant after centrifugating the loaded AuNPs, and on the supernatants after the release process. The optimised loading percentage is (41 ± 4)%, calculated in triplicates. The release is found to be above 50% after 3 hours.

#### 2.2.4 Photothermal characterisation

The PT properties of AuNPs-3MPS-DEA were investigated by using a thermo-optical setup, equipped with a Continuous Wave (CW) diode laser (Coherent Powerline) operating at 532 nm (green laser) and a high-resolution thermal camera (FLIR, A655sc) for measuring the time/temperature profiles under lateral pumping laser irradiation. The camera produces thermal images of 640 × 480 pixels with an accuracy of ± 0.2 °C.

The thermal camera integrates with the proprietary (FLIR ResearchIR Max) software, which is used to record the thermal data acquired by the camera and process them into temperature values. Temperature values are measured in an elliptical region of interest (ROI) of 0.084 cm^2^.

Laser light irradiations were performed for two minutes utilising power density values in a range from 3.5 W cm^−2^ to 17.8 W cm^−2^ and utilising a cooling time of 30 s.

The temperature values measured during the cooling time were utilized to evaluate the PT conversion efficiency (*η*) according to the Roper model^25^ as described in the following equation:1
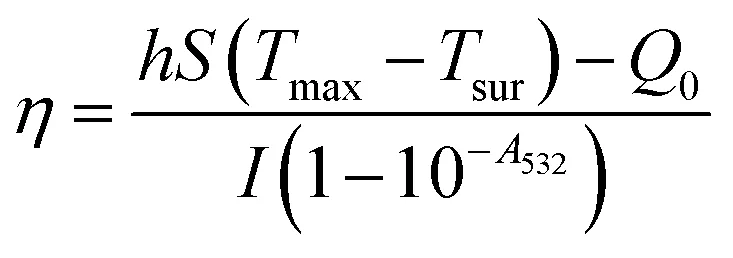
In eqn (1), *h* is the heat transfer coefficient, and *S* is the surface area of the quartz cuvette covered by the sample. *T*_sur_ and *T*_max_ are the ambient temperature and maximum temperature reached under laser irradiation, respectively. *Q*_0_ represents the heat dissipated due to solvent absorption under laser irradiation and *I* and *A*_532_ are the incident laser power density and absorbance of the solutions at 532 nm, respectively. The product *h* × *S* can be calculated as follows:2
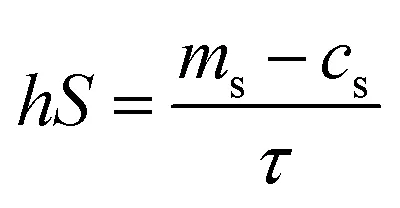
where *m*_s_ and *c*_s_ are the mass and heat capacity of the dispersion, respectively and *τ* is the time constant value. As *τ* is inversely proportional to *h* × *S*, it follows that *τ* is a useful parameter to evaluate *η*. To obtain *τ*, the dimensionless driving force temperature (*θ*) is introduced.3
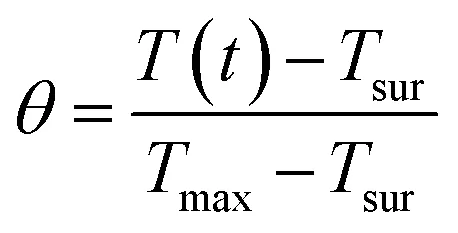
By elaborating eqn (3) it results that:4
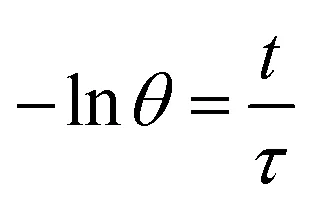


Hence, *τ* can be calculated as the negative reciprocal of the slope obtained by plotting ln(*θ*) *versus t* considering the temperature values recorded during cooling time (*t*).

#### 2.2.5 UV-visible absorption

UV-visible spectra were recorded using a Varian Cary 100 (wavelength range of 190–900 nm) UV-Vis spectrophotometer at room temperature. Quartz cells with a 1.5 mL volume and 1 cm optical path length were used.

#### 2.2.6 FT-IR: transmission setup and acquisition settings for silicon deposition

100 µL of AuNPs, AuNPs-TZ and TZ solutions were dropped on the high-resistivity silicon substrate and left for evaporation at room-temperature under inert conditions.

IR absorption spectra of samples deposited on high-resistivity silicon (HR-Si) substrates were collected using a Bruker Vertex 70v operating in transmission mode. Spectroscopic measurements were carried out at room temperature (22 °C) and under vacuum conditions to minimise interference from water vapour and CO_2_ absorptions. For each sample, several repetitions of 128 scans were recorded over the 50–6000 cm^−1^ spectral range with a spectral resolution of 4 cm^−1^. The HR-Si reference spectrum was collected immediately prior to each sample measurement, using the same experimental settings. The spectra analysis was performed using OPUS 8.2 software (Bruker Optics) and MATLAB-based algorithms (ver. 2024b, MathWorks Inc., Natick, MA, USA) for absorbance calculation, baseline correction, cut and average. The analysis was focused on the spectral ranges between 1490–1750 cm^−1^ and 2800–3000 cm^−1^, *i.e.* where the Amide I band of proteins^26,27^ and vibrations of C–H bonds associated with thiols and TZ are located,^28,29^ respectively. Each spectrum was deconvoluted into its spectral components using the 2nd-derivative minima analysis to obtain the spectral frequencies for subsequent multiple Gaussian fitting performed with OPUS 8.2 software 26.^30,31^ The residual mean square error (RMSE) was used as the goodness-of-fit parameter.

#### 2.2.7 FTIR: ATR setup and acquisition settings for suspensions

IR absorption spectra of suspensions of 0.25 mg mL^−1^ AuNPs, AuNPs-TZ and TZ were collected in the same experimental conditions as described above (in vacuum and at 22 °C), equipped with an ATR–Diamond single reflection module. The samples were exposed to a CW laser beam with *λ* = 532 nm and 90 mW power. Drops of 5 µL from the three samples were collected before and after the laser exposition, directly dropped on the ATR diamond crystal for each ATR-IR measurement, collecting several repetitions of 128 scans between 400–4000 cm^−1^ with a spectral resolution of 2 cm^−1^. Water reference spectra were collected immediately prior to each sample measurement and put a water drop of 5 µL on the diamond, using the same experimental settings to perform a background subtraction. OPUS 8.2 software (Bruker Optics) and MATLAB-based algorithms (ver. 2024b, MathWorks Inc., Natick, MA, USA)^28,29^ were used to perform the spectral analysis: absorbance calculation, baseline correction, water subtraction, and ATR advanced correction.

#### 2.2.8 Dynamic light scattering

The hydrodynamic diameter (<2*R*_H_>, nm) of AuNPs in dichloromethane was measured on a Malvern Zetasizer Nano ZS90 instrument at 25 °C using a 4 mW laser light with the wavelength of 632.8 nm and an automatic attenuator. Measurements were done in triplicate and reported as mean ± standard deviation.

#### 2.2.9 Small angle X-ray scattering

Small angle X-ray scattering (SAXS) measurements were conducted in transmission geometry at beamline BL2 of the DELTA synchrotron light source in Dortmund, Germany.^32^ The experiment was performed at a photon energy of 12 keV and with a beam size of 0.5 × 0.5 mm^2^. SAXS patterns of solutions in glass capillaries (Hilgenberg, Malsfeld, Germany) with 1.5 mm diameter were recorded using a large-area MAR345 (marXperts GmbH, Norderstedt, Germany) detector. Calibration measurements were performed using a silver behenate standard to refine the geometrical parameters of the setup. To determine the scattering background, a capillary filled with pure buffer was measured. Temperature measurements were conducted using a temperature cell (Linkam Scientific Instruments Ltd) with a heating/cooling rate of 1 °C min^−1^. Thermal equilibration was allowed for 5 minutes before each measurement. Depending on the sample examined, exposure times between 60 s and 600 s were selected.

## 3. Results and discussion

The nanosystem used in this study, AuNPs-3MPS-DEA, has proven its usefulness in drug delivery of both a small drug (MTX) and an antibody (TZ), with anti-cancer purposes, as demonstrated in recent literature.^23,24^ To assess the possibility of employing the pristine AuNPs in PTT, they were herein studied with PT analysis. Furthermore, the structural features of the AuNPs and Trastuzumab system and the molecular interactions underlying their interaction were investigated with a series of complementary characterisation techniques, with the aim of obtaining a comprehensive understanding of the loaded system. FT-IR spectroscopy was employed to investigate the interfacial chemistry between AuNPs and the antibody and investigate the molecular interaction between surface thiol groups and Trastuzumab. Complementary, the PT behaviour, coupled with FT-IR analyses, provided insights into possible future use of this system for controlled release exploiting the photothermal effect. To investigate the loading and release kinetics of TZ from the surface of the nanoparticles, SAXS was used to monitor both processes over time.

### 3.1 PT analysis of AuNPs in colloidal dispersion

The PT characterisation of AuNPs was carried out by irradiating 1 mL of the sample for 120 s followed by a cooling time of 60 s. The irradiation steps were performed at different laser power densities, in the range from 3.5 W cm^−2^ to 17.8 W cm^−2^. The experimental data derived from the thermal images are reported in Fig. 1a as mean temperature as a function of irradiation time inside an elliptical ROI.

**Fig. 1:**
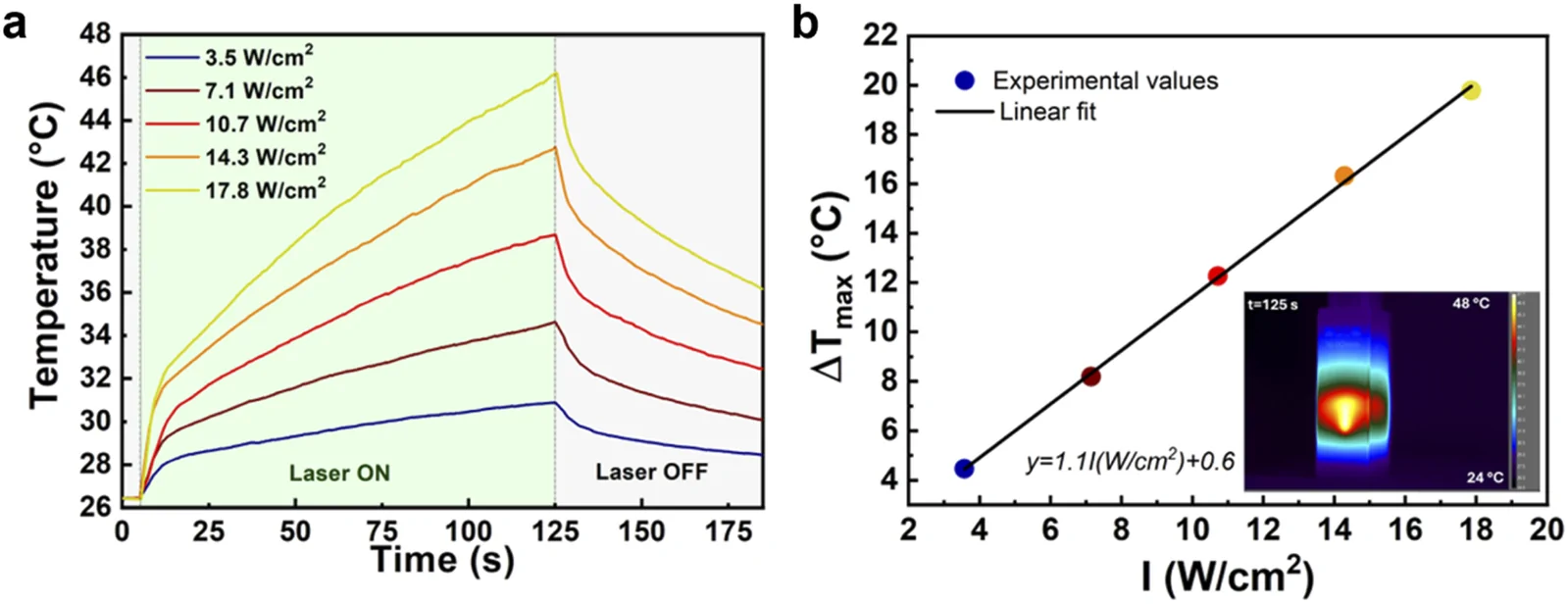
PT investigation on a colloidal dispersion of AuNPs-3MPS-DEA. (a) Time – temperature profile obtained reporting the maximum temperature measured in the ROI as a function of the irradiation time at different laser power density. (b) Variation of the mean temperature values measured at the end of the irradiation time, as a function of laser power intensity. The inset shows a PT images captured just before shutting down the laser at the power density of 17.8 W cm^−2^.

Furthermore, the CW laser represents an appropriate choice for PT as it avoids the detrimental risk of NPs melting or reshaping, which may occur when femtosecond or picosecond laser are used as light sources.^33,34^

AuNPs-3MPS-DEA colloidal dispersion (0.30 mg mL^−1^) shows an absorption peak centred at 516 nm (absorbance 2.5, see Fig. S1),^23,24^ accordingly the CW 532 laser has been selected to perform the PT characterisation. This wavelength is widely used in dermatology,^35,36^ and the integration of AuNPs in 532 nm laser treatments highlighted promising efficacy for cutaneous vascular lesions^37^ and hyperpigmentation.^38^

The temperature values increase with the increase of the laser power density, irrespectively of the employed laser power density, as the light source is switched on a rapid and continuous increase of the temperature is observed especially for the first 10 s of irradiation. Indeed, by plotting the maximum temperature increase as a function of laser power density, a linear dependence can be found, as shown in Fig. 1b resulting in a *R* square value of 0.99. Therefore, this gold nanoparticles system exhibits a PT behaviour in full agreement with the literature.^39^

Following the Roper model, the photothermal efficiency was evaluated, calculating a time constant value *τ* equal to 33 s for the laser power density of 3.5 W cm^−2^ corresponding to a laser power of 0.3 W.

Jiang *et al.*^39^ performed a similar PT characterisation experiment by irradiating 1 mL of 4.35 nm AuNPs under a 532 nm laser (0.3 W) giving rise to *τ* value of 218 s (87 s considering the dilution factor). As a result, it can be inferred that the heat transfer efficiency of AuNPs-3MPS-DEA is higher with respect to citrate capped AuNPs with a similar size. This difference could be ascribed to the capping agents, as the thiol ligands generate a more compact stabilising layer around AuNPs, due to the unique Au–S covalent interaction, stronger than the electrostatic interaction obtained with citrate ions.^40,41^ Similar to silica shells,^42,43^ this compact layer^44^ could reduce thermal resistance at nanoparticle–water interface thus enhancing heat dissipation, with respect to citrate-stabilised AuNPs.

Overall, this AuNPs system exhibits promising photothermal properties. These results indirectly indicate that 3MPS and DEA as a functionalising layer minimise AuNPs aggregation, improving stability and preserving a high absorption cross-section, ensuring efficient light-to-heat conversion ability.

### 3.2 FT-IR spectroscopy and photothermal analysis of TZ-loaded AuNPs

To understand and investigate the interfacial chemistry between the AuNPs surface, *i.e.* the functionalising thiols, and the antibody TZ, pristine AuNPs, TZ and AuNPs-TZ were studied with FT-IR spectroscopy, both in the solid state and in the dispersed state. Moreover, FT-IR measurements were coupled with CW laser illumination at 532 nm, to evaluate the structural and interfacial shifts due to the photothermal effect, comparing it to pristine gold nanoparticles (*cfr*. 3.1).

#### 3.2.1 Samples on HR-Si substrates

FT-IR spectra recorded in transmission mode of TZ, functionalised AuNPs and AuNPs-TZ are displayed in Fig. 2. The main differences between the absorption curves are observed in the low wavelength range (1000–1500 cm^−1^), in the amide bands (between 1500–1750 cm^−1^) and in the C–H bonds vibrational range (2800–3000 cm^−1^).

**Fig. 2:**
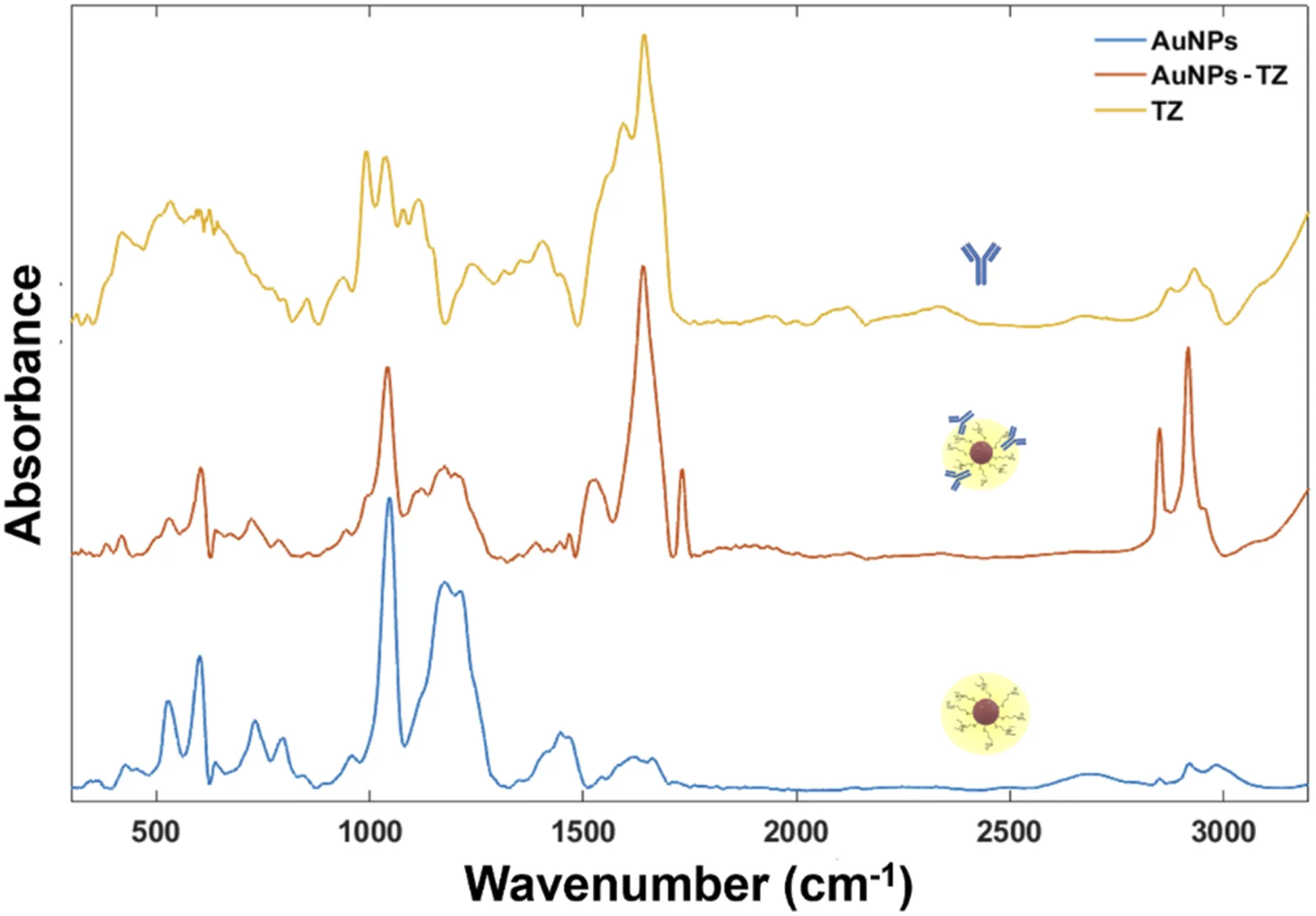
AuNPs (blue curve), AuNPs-TZ (red curve) and TZ (yellow curve) IR absorption spectra are reported. The spectra were shifted for better visualisation.

In the 1000–1500 cm^−1^ region, TZ (yellow curve) exhibits characteristic protein vibrational modes. Peaks associated with TZ vibrations can be observed at 991, 1034, 1074, 1119, 1146 cm^−1^.^45^ Conversely, AuNPs and AuNPs-TZ (blue and red curves, respectively) show different peaks at 1045, 1173 and 1215 cm^−1^, attributable to thiols vibrations.^23,28^ These bands can be assigned to vibrational modes of SO_3_^−^, a functional group present in 3MPS. This observation suggests that, remaining detectable after protein loading, 3MPS might not directly participate in the protein-nanoparticle binding. We therefore infer that these vibrational modes are enhanced by protein attachment and remain detectable in the spectra.

The spectral range between 1500 and 1700 cm^−1^ corresponds to Amide I and II bands, which include vibrational modes due to C=O and C–N bonds. As shown in Fig. 2, both TZ and AuNPs-TZ spectra exhibit a strong absorbance in this range, indicating that the contribution of AuNPs alone is minor in this spectral region. Fig. 3 shows the deconvolution of the TZ and AuNPs-TZ spectra in the 1500 and 1750 cm^−1^ range, while Fig. S2 reports the deconvolved Amide I spectra for each sample.

**Fig. 3:**
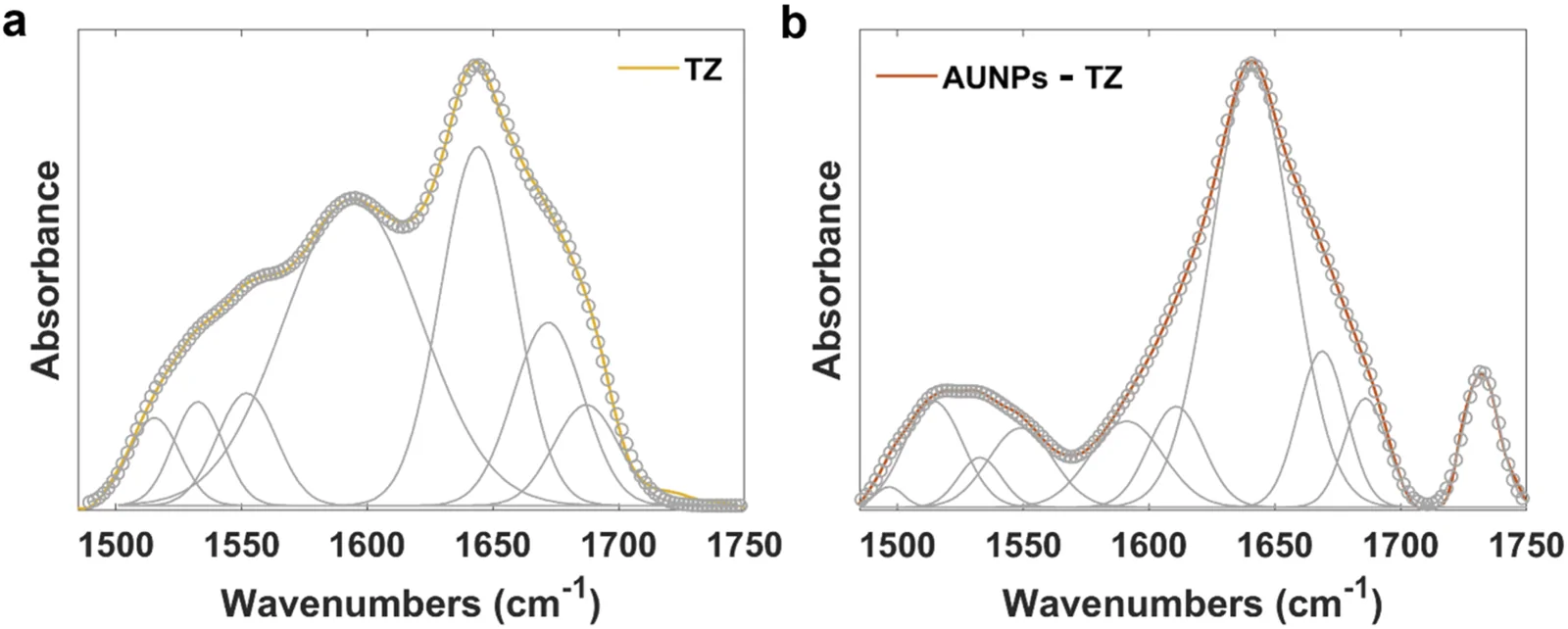
TZ (a) and AuNPs-TZ (b) Amide I IR spectra are shown with the deconvoluted gaussian bands (light grey lines) and the fitted spectrum (light grey empty dots).

Both TZ and AuNPs-TZ spectra display the same gaussian component at 1515, 1533, 1550, 1644, 1670 and 1687 cm^−1^. The most significant differences arise from the presence of the peak at 1732 cm^−1^ in the AuNPs-TZ and a peak at 1591 cm^−1^ in the TZ spectrum. Notably, the latter is the largest one and is completely absent in the AuNPs-TZ spectrum. This peak presents a peculiar shape and position, as it lies between the Amide I (1600–1700 cm^−1^) and the Amide II (1500–1600 cm^−1^) range.

A similar absorption can be ascribed to the presence of protein aggregates in solution, which may occur when the protein concentration is too high relative to the solution volume. In our case, several drops of protein solution were deposited on the HR-Si surface and left to dry. We assume that, before complete solvent evaporation at room temperature, the protein concentration in the drop increased as the drop volume decreased, causing aggregation phenomena.

On the other hand, despite having the same concentration, this behaviour does not appear in the AuNPs-TZ spectra, suggesting that proteins were effectively stabilised on the nanoparticles surfaces in aqueous solution and did not aggregate during the drying process.

By observing the spectral range between 2800 and 3000 cm^−1^, Fig. 2b shows that the AuNPs-TZ spectrum exhibits an enhanced absorbance with respect to both AuNPs and TZ, associated with the characteristic absorption features of CH_2_ and CH_3_ groups.

The spectral deconvolutions of the three samples are reported in Fig. S3 in SI. AuNPs-TZ spectrum (red curve) displays two main peaks at 2851 and 2917 cm^−1^ corresponding to the symmetric and antisymmetric CH_2_ stretching modes of AuNPs. The same peaks are also observed in the AuNPs spectrum (blue curve).

#### 3.2.2 Suspended samples

To analyse the PT behaviour of the nanoparticles, three different samples were prepared as colloidal dispersions: AuNPs, AuNPs-TZ and TZ. The study was focused on two different steps: firstly, the ATR-IR spectra of all three pristine samples were collected; secondly, they were collected after irradiation at 532 nm at 90 mW (*cfr.* 3.1) with exposure times up to 15 minutes.

In Fig. 4, ATR-IR spectra of AuNPs, AuNPs-TZ and TZ in suspension recorded before and after exposure to a 532 nm laser are displayed and compared.

**Fig. 4:**
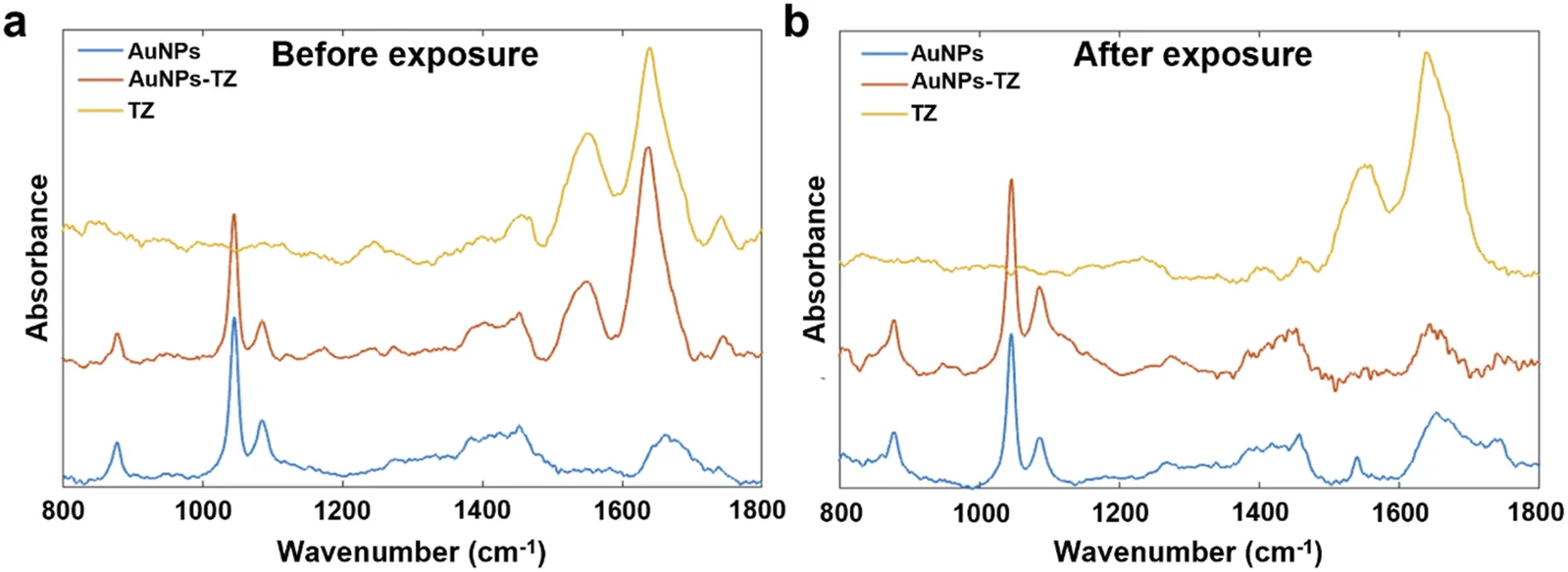
AuNPs (blue curve), AuNPs-TZ (red curve) and TZ (yellow curve) IR spectra between 800–1800 cm^−1^ range are reported with before (a) and after (b) laser exposure (15 minutes). The IR spectra were shifted for visualisation.

By analysing the three spectra acquired before laser exposure, characteristic features associated with protein and thiols can be identified. These features are the same as those observed in the deposited samples. In particular, similarly to what was observed in the analysis of samples deposited on HR-Si substrate, thiol groups present in the AuNPs and AuNPs-TZ samples exhibit absorption peaks at low wavenumbers, specifically at 1045 and 1085 cm^−1^, which can be attributed to SO_3_^−^ vibrational modes.^23,28^ Meanwhile, in the spectral ranges 1500–1600 cm^−1^ and 1600–1700 cm^−1^, the AuNPs-TZ and TZ samples show the Amide II and Amide I bands, respectively.^46^

By comparing these absorption bands with those observed in the deposited samples, it is evident that the Amide I and Amide II bands of TZ undergo a conformational change, adopting a spectral shape similar to that observed in the AuNPs-TZ samples. This observation confirms our hypothesis regarding the aggregation phenomenon in the solid sample, and supports the idea that, in the AuNPs-TZ solid system, proteins interact with the surface functionalising layer while preserving their structural conformation.

Focussing on the differences observed in the IR spectra recorded after the exposure to light at 532 nm, we can easily note that AuNPs-TZ spectrum completely loses the peaks associated with the TZ protein. This represents the most significant difference observed and the one of primary interest in this study and gives a preliminary indication of protein detachment, as the vibrational peak is enhanced by the proximity to the gold surface, therefore when the protein detaches the peak is not as pronounced as it was while interacting with the surface of the AuNPs.^47^

The resulting AuNPs spectrum is very similar to that of AuNPs, suggesting that the sample no longer contains protein-related features (1600–1700 cm^−1^ Amide I band). This indicates that the exposure to the gold plasmonic resonance induces an excitation of the gold nanoparticles leading to protein detachment.

Therefore, the exposure time was varied to observe the spectral behaviour. Fig. 5a collects all the IR spectra acquired at different exposure times, from 0 to 15 minutes, while Fig. 5b shows the IR spectra acquired at increasing temperatures, up to 40 °C.

**Fig. 5:**
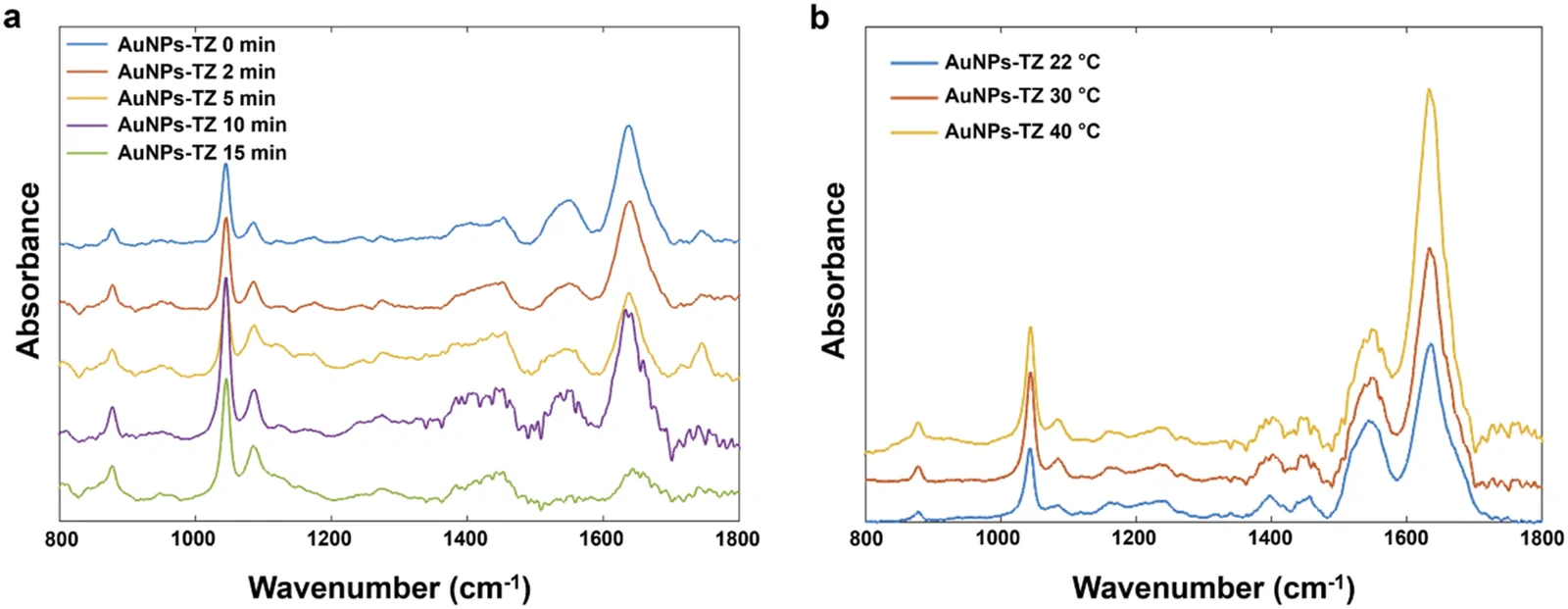
a) AuNPs-TZ IR spectra between 800–1800 cm^−1^ range changing the exposure time; (b) AuNPs-TZ IR spectra between 800–1800 cm^−1^ range increasing the sample temperature (22 °C, 30 °C and 40 °C). The spectra are shifted for visualisation.

A mean value of the exposure energy absorbed by each sample can be estimated from the laser power output and the laser exposure duration, reported in Table S1 in the SI. The same energy used in the photothermal analysis (*cfr.* Section 3.1) was applied by exposing the sample to the laser for more than 15 minutes at 4.5 W cm^2^; subsequently, the exposure time was decreased. From the infrared spectra, it is evident that with 5 minutes of exposure the sample does not exhibit any significant modifications. When the exposure time was increased to 10 minutes, changes can be observed in the Amide I band shape. The corresponding curve (purple line in Fig. 5a) appears to be affected by increased noise in the Amide I region. This behaviour suggests that the protein detachment process may be initiating at this stage.

To ensure that the protein detachment was attributable only to nanoparticle excitation, the behaviour of the suspensions under temperature variation was studied. A temperature increase of more than 5 °C was investigated, corresponding to the temperature rise measured during the photothermal effect induced by the laser. Starting from room temperature (22 °C), the temperature was increased up to 40 °C, while monitoring the behaviour of AuNPs-TZ. Fig. 5b shows the spectra of the suspended sample recorded at 30 °C, 40 °C, and room temperature.

The three spectra exhibit the same spectral shapes. In particular, all of them present the Amide I peak. The spectrum of the sample exposed to 40 °C presents a behaviour with more noise, but the peak is strongly visible and present. This observation supports our hypothesis that protein detachment is linked to gold nanoparticle plasmon excitation rather than to a simple temperature increase.

### 3.3 SAXS study, loading and release

The kinetics of loading and release of TZ on the surface of AuNPs were studied with SAXS studies. By following the scattering profile of AuNPs and its changes over time during the loading and release processes, the shifts in the packing of gold nanoparticles in the different stages of loading and release can be studied.

As demonstrated in our previous paper, the loading of TZ on AuNPs-3MPS-DEA caused some degree of aggregation, due to the interactions of big molecules like an antibody with the surface of the gold nanoparticle;^24^ herein, we followed this effect more accurately and how it evolved in time under the experimental parameters. SAXS was employed to study the system firstly as pristine gold nanoparticles, then the kinetics of the loading and release of TZ on the surface was followed, measured at 1 hour, 2 hours and 3 hours. The scattering profile of pristine nanoparticles (shown in Fig. 6, top left) was fitted with a spherical model and the gyration radius of the nanoparticles was found to be (2.43 ± 0.12) nm (size distribution profile shown in Fig. S4). It is noteworthy that this value pertains to the gold core, as the density contrast of the thiol shell to the water phase has minor influence on the scattering intensity compared to the gold contrast.

**Fig. 6:**
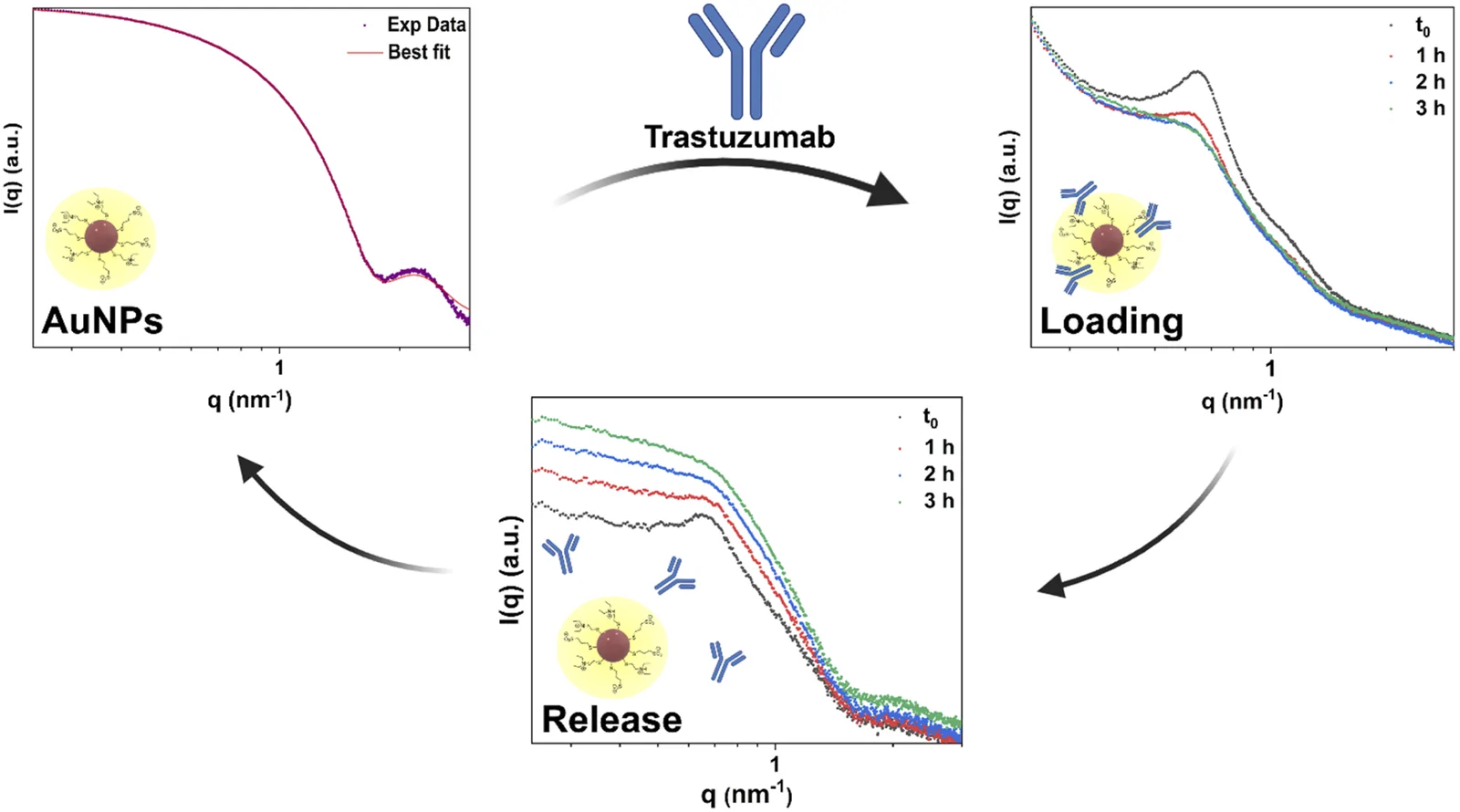
SAXS profiles of AuNPs and AuNPs-TZ; top left: pristine AuNPs with the best fit superimposed; top right: loading kinetics of TZ on AuNPs; bottom: release kinetics of TZ from AuNPs.

As TZ is added to the nanoparticles, the scattering profile changes, exhibiting a correlation peak at *q*_max_ = 0.7 nm^−1^ (*t*_0_, grey dots in Fig. 6, top right), which is accompanied by a strong increase in scattering intensity for *q* → 0.; this indicates a sharp increase in attractive interparticle interactions, which in this case are mediated by the presence of the antibody which interacts with the surface of multiple gold nanoparticles, producing this aggregation effect. Using the simple approximation *D* = 2π/*q*_max_, we obtain a particle spacing *D* of approximately 9 nm, compatible with antibody-mediated bridging between the nanoparticles.^48^ With time a steady decrease in scattering intensity can be observed across the entire *q* range, which can be explained by sedimentation of the larger aggregates (the beam size is significantly smaller than the capillary diameter). After 1 hour, and increasingly after 2 and 3 hours (respectively, red, blue, and green dots), this effect is mitigated, with the system gradually reaching equilibrium. While some degree of aggregation is still visible, as a shoulder is still present at 0.7 nm^−1^, this is to be expected and indicates interaction between the biomolecule and the surface thiols of the gold nanoparticles.^24,49^

The bottom graph in Fig. 6, shows the release over time at 37 °C; at *t*_0_ the curve is similar to the one after the loading shown in the top right graph, whereas an increase in intensity over time is observed, as well as a decrease in the strength of the correlation peak, which can be explained by a dissolution of the aggregate structures. However, it should be noted that aggregation occurs much faster (the formation rate of the aggregates is outside the time window of the measurement) than the release of the antibodies, which is still not completely finished even after 3 hours, as already found in our last study on this system.^24^

The SAXS study allowed to evaluate the kinetics of the loading and release processes for AuNPs-TZ; by confirming previous scientific findings and exploring new time-based dynamics of the loading and release processes, new perspectives for this system can be envisioned, especially in the controlled release of the drug from the AuNPs surface.

## 4. Conclusions

AuNPs-3MPS-DEA loaded with TZ were characterised in order to assess their potential use in PTT and PT-assisted drug release. Pristine AuNPs showed great promise as PTT probes, with a high cross-section and thus a high temperature increase (up to 20 °C) when irradiated with 532 nm CW laser; notably, the photothermal behaviour of this thiol functionalised system is more promising if compared with citrate stabilised AuNPs.

FT-IR studies allowed to confirm that no conformational change occurs in TZ when loaded on the surface of the AuNPs and that the vibrational bands pertaining to the amides in the protein shift upon loading with the nanoparticles, suggesting their involvement through weak, non-covalent interactions in the binding to the thiol functionalised gold surface. Moreover, to assess the possibility of a controlled release of TZ from the surface, FT-IR studies were coupled with the excitation of the AuNPs at 532 nm with a CW laser, resulting in a release of the antibody from the surface upon illumination of the sample.

Lastly, the kinetics of the loading and release processes, investigated through SAXS experiments in time, confirmed our previous findings, *i.e.* that 3 hours is the optimal time to reach the equilibrium for the loading, and in the same time frame, a complete release can be obtained.^24^

This study led us to understand the chemical interactions at the interface between thiol functionalised AuNPs and bioactive molecules, together with the exploration of further potential therapeutic tools. These results provide a solid framework, combining PTT and controlled release, from which future therapeutic strategies in nanomedicine can be explored.

## Conflicts of interest

There are no conflicts to declare.

## Supplementary Material

RA-016-D6RA07147C-s001

## Data Availability

The data supporting this article have been included as part of the supplementary information (SI). Supplementary information is available. See DOI: https://doi.org/10.1039/d6ra07147c.
