# Supplementary material for: Mixed-thiol functionalised gold nanoparticles for trastuzumab delivery: chemical interaction and photothermal therapeutic potential
Source: RSC Adv. 2026 Oct 14;16(47):62953–61. doi: 10.1039/d6ra07147c (PMC13667367; doi:10.1039/d6ra07147c)
Supplement: Supplementary file 1 [file RA-016-D6RA07147C-s001.pdf]

Supplementary Information (SI) for RSC Advances.
This journal is © The Royal Society of Chemistry

Supporting Information

Mixed-thiol Functionalised Gold Nanoparticles for Trastuzumab Delivery: Chemical Interaction and Photothermal Therapeutic Potential

Tommaso A. Salamone[1,2,*], Rosanna Mosetti[3,4], Annalisa D'Arco[4], Stefano Lupi[4], Francesca Petronella[5], Arianna Avitabile[6], Flaminia Pompeo[6], Luciano De Sio[6], Sofia Marotta[1], Stella Mrmić[1], Beatrice Pennacchi[2], Sara Cerra[2], Souren Grigorian[7], Michael Paulus[8], Maurizio Alimandi[1], Pankaj Trivedi[9], Eleni Anastasiadou[1], Ilaria Fratoddi[2,*]

[1] *Department of Clinical and Molecular Medicine, Sapienza University of Rome, 00189, Rome, Italy*

[2] *Department of Chemistry, Sapienza University of Rome, 00185, Rome, Italy*

[3] *Department of Basic and Applied Sciences for Engineering, Sapienza University of Rome, 00161 Rome, Italy*

[4] *Department of Physics, Sapienza University of Rome, 00185, Rome, Italy*

[5] *National Research Council, Department of Chemical Sciences and Materials Technologies, Institute of Crystallography, 00010, Montelibretti, Italy*

[6] *Department of Medico-Surgical Sciences and Biotechnologies, Sapienza University of Rome, 04100, Latina, Italy*

[7] *Institute of Physics, University of Siegen, D-57068, Siegen, Germany*

[8] *Fakultät Physik/DELTA, Technische Universität Dortmund, 44221 Dortmund, Germany*

[9] *Department of Experimental Medicine, Sapienza University of Rome, 00161, Rome, Italy*

Corresponding authors: Tommaso.salamone@uniroma1.it; ilaria.fratoddi@uniroma1.it

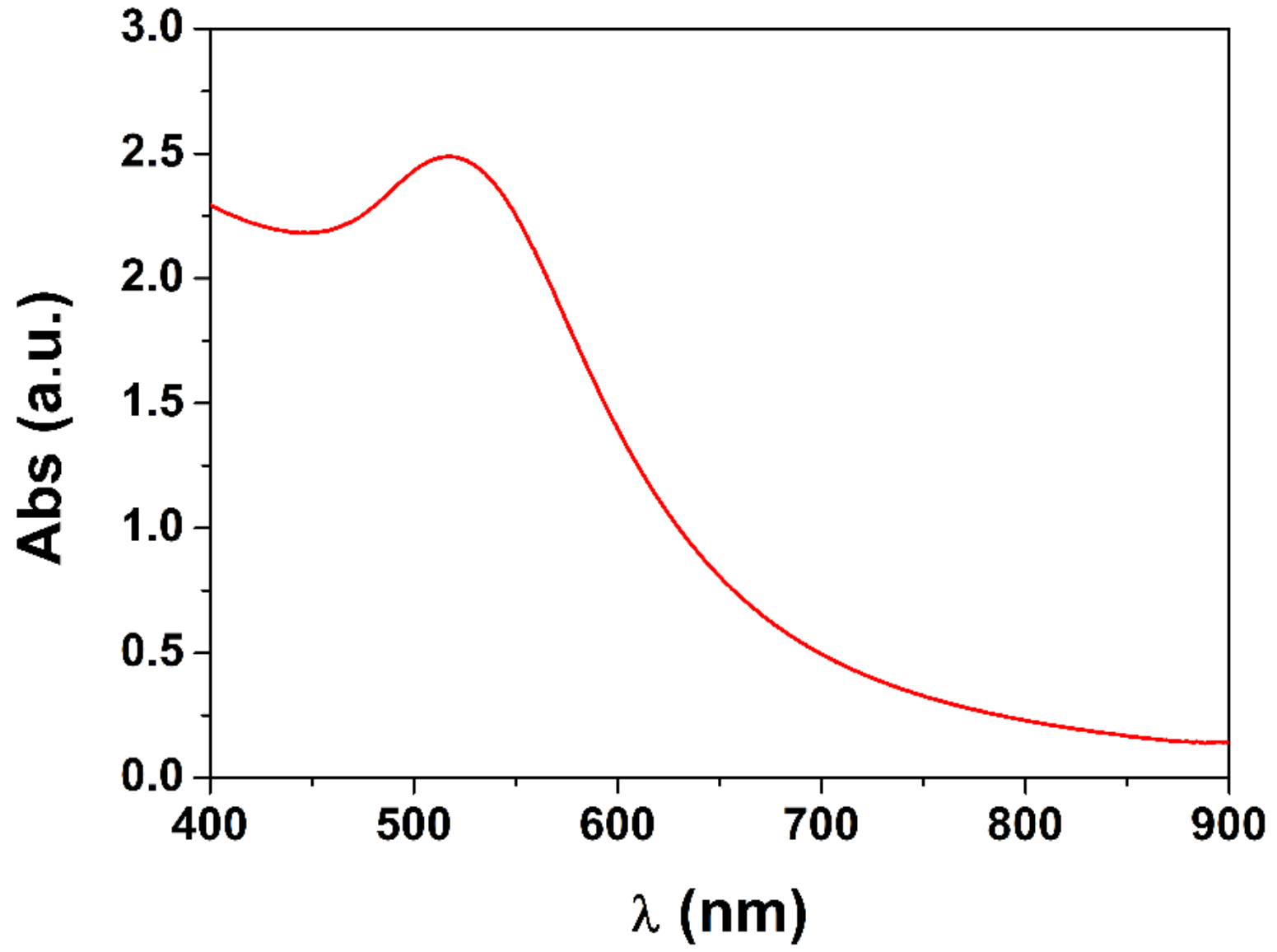

Figure S1. UV-vis spectrum of AuNPs at 0.3 mg/mL concentration in water. λ$_{max}$ = 516 nm, Abs_{532} = 2.44.

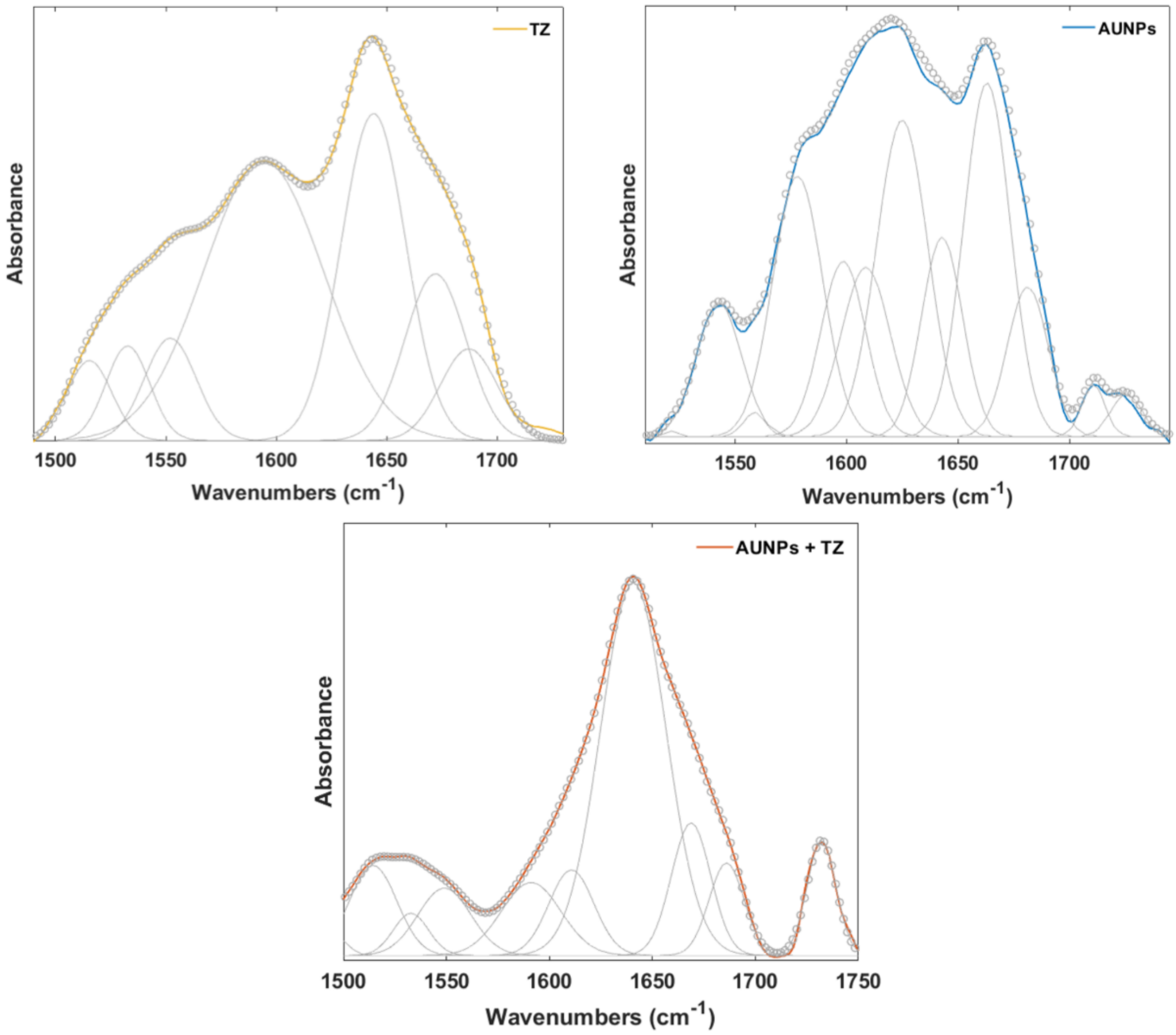

Figure S2. TZ (yellow curve), AuNPs (blue curve) and AuNPs-TZ (red curve) Amide I IR spectra are shown with their deconvoluted gaussian bands (light gray lines) and the fitted spectrum (light gray empty dots).

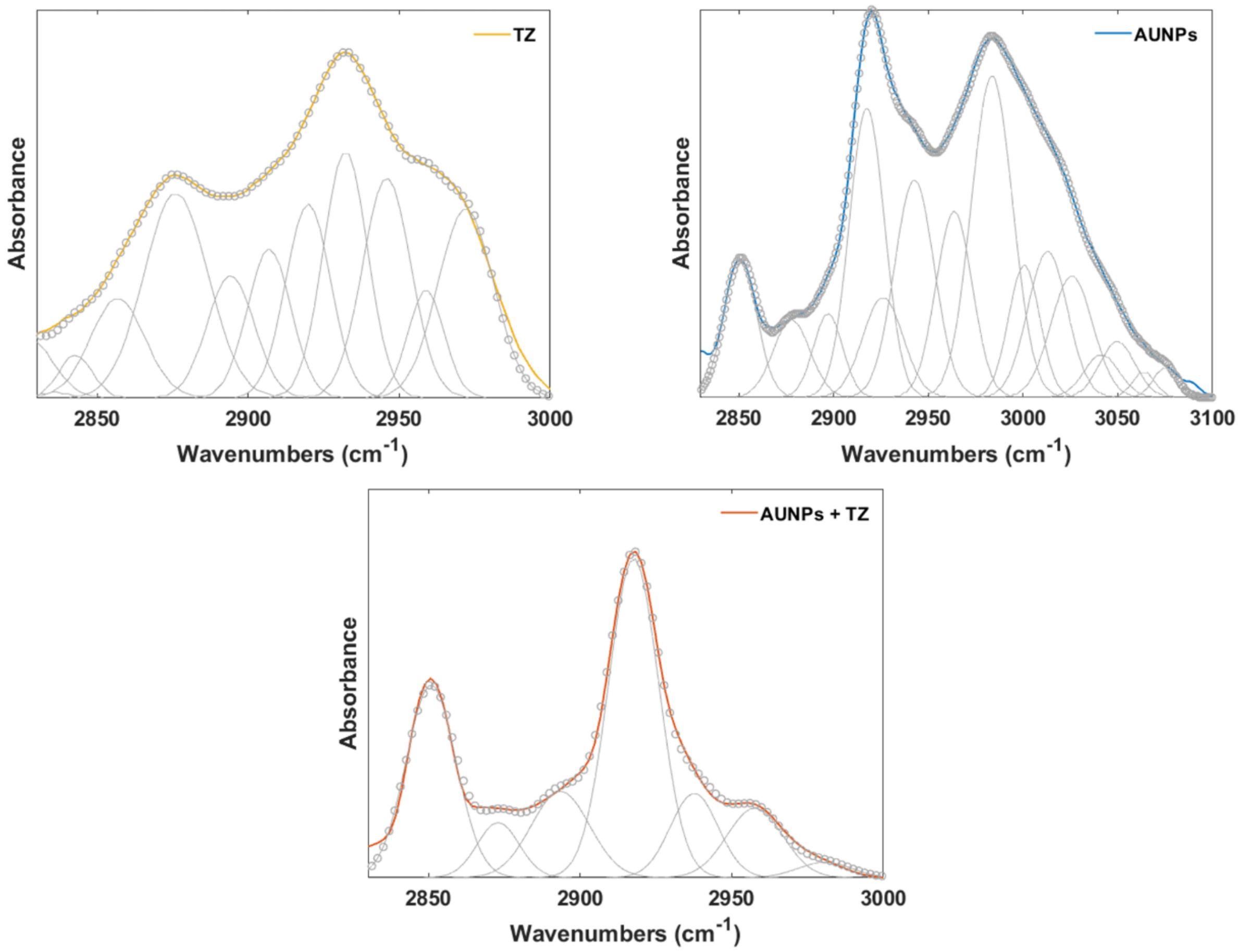

Figure S3. AuNPs (blue curve), AuNPs-TZ (red curve) and TZ (yellow curve) IR spectra between 2800-3000 cm^{-1} range are reported with their deconvoluted gaussian bands (light grey lines) and the fitted spectrum (light grey empty dots).

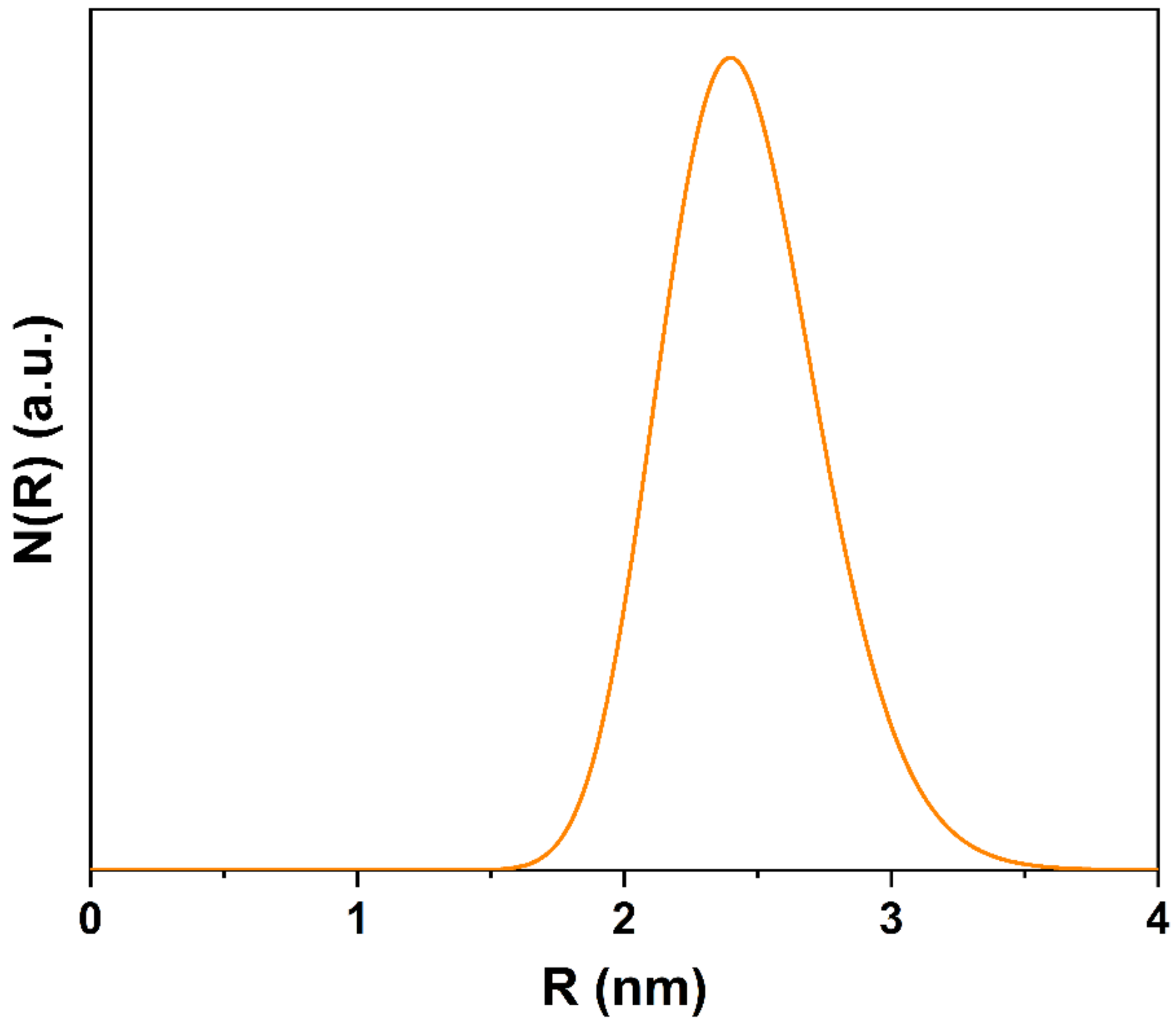

Figure S4. Size distribution (gyration radius) of pristine AuNPs derived from the fit of the SAXS profile.

Table S1. Mean value of the exposure energy absorbed by AuNPs-TZ samples exposed to a continuous Wave (CW) laser beam with λ = 532 nm and 90 mW power

Exposure time (min)	Energy (J)
0	0
2	10.8
5	27
10	54
15	81
